# Vibrio vulnificus Necrotizing Fasciitis in Upper Limbs and Septicemia Following Pinch Injury by Mud Crab: A Case Report

**DOI:** 10.7759/cureus.24393

**Published:** 2022-04-22

**Authors:** Asadarng Phitsamai, Worawong Chueansuwan, Dhitiwat Changpradub

**Affiliations:** 1 Infectious Disease Division, Internal Medicine Department, Phramongkutklao Hospital, Bangkok, THA

**Keywords:** mud crab, pinch injury, necrotizing fasciitis, septicemia, vibrio vulnificus

## Abstract

*Vibrio vulnificus* necrotizing fasciitis is a rare emergency and has a high mortality rate condition occurring among patients with cirrhosis, iron overload states, chronic renal failure, malignancy, HIV, or immunosuppressive medications. Here, we report a case of nonfoodborne *Vibrio* infection caused by *V. vulnificus* presenting as bilateral necrotizing fasciitis on the hands and lower arms after a pinch injury by a mud crab in a 64-year-old man with hypertension presenting with acute fever, bilateral hand swelling, and pain. The patient was treated with emergency fasciotomy and intravenous antibiotics. The outcome of such cases depends on early diagnosis and appropriate surgical and medical management.

## Introduction

*Vibrio vulnificus*, a halophilic, curved, motile gram-negative bacilli, is naturally occurring in warm estuarine waters [1]. In the US, approximately 200 infections occur yearly, especially during the summer months after ingesting raw seafood [1]. From 1988 to 1996, *Vibrio* surveillance 189 of 422(45%) infections with reporting on the wound type infections and 204 of 422(48%) followed the ingestion of seafood present with primary septicemia or gastroenteritis. In this report, wound infections were fatal in 17% of cases [2].

Nonfoodborne vibrio infections (NFVIs) reported the most symptoms and signs, including fever, cellulitis, and bullae [3,4]. NFVIs associated mortality was 78%, and the risk factors of patients with NFVIs were handling seafood (58%), swimming (54%), boating (40%), walking (34%), and bite (12%) in the United States [3]. Regarding the high prevalence of underlying liver disease and other chronic diseases including diabetes mellitus, alcoholism without specific liver disease, cancer, renal disease, gastrointestinal disease, steroid-dependent rheumatoid arthritis, and hematologic disorders associated with immunosuppression [1,3,5,6]. Most patients who have wound infection developed symptoms within 24 hours, and case-fatality rates increased with greater delays in antibiotic treatment [6]. The keys to survival were early diagnosis and treatment including surgical and medical treatment [7,8].

## Case presentation

**Figure 1:**
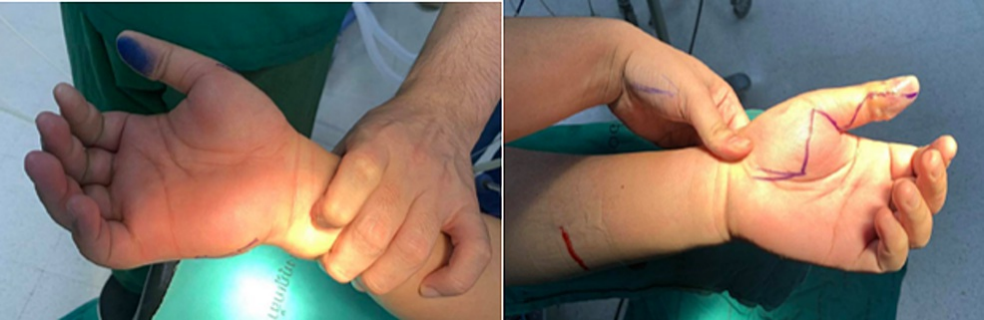
Picture of the patient’s hands with multiple tense bullae and edema developed within 13 hours.

**Figure 2:**
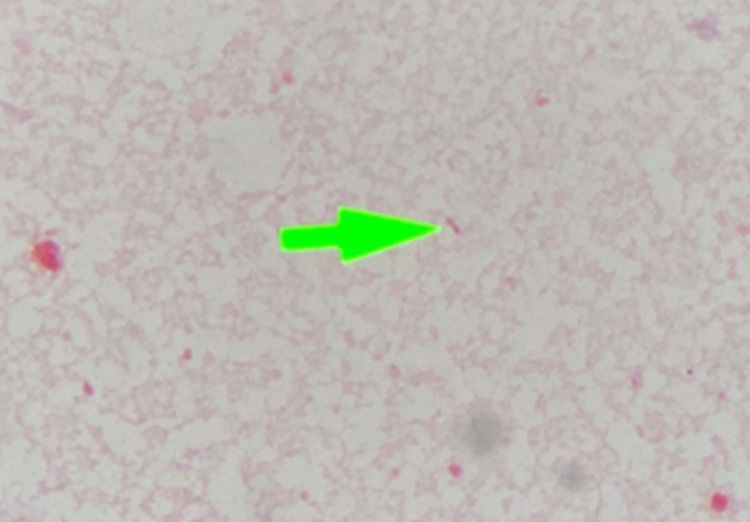
Tissue gram stain indicating rare gram-negative bacilli.

A 64-year-old Thai male with an underlying hypertension who developed bilateral hand swelling and pain for three hours before admission in June 2021. Thirteen hours earlier, he had been pinched by a serrated mud crab on his left thumb and right ring finger while he was going to release them in a mangrove forest. Ten hours later, he developed a sudden onset of dull aching pain and swelling on his hands, and his pain score was 8 of 10. He presented with high-grade fever, shivering, and tachypnea. Therefore, he decided to go to the primary hospital near his home. His physical examination was remarkable, with a temperature of 37.9°C, a pulse rate of 140/min, blood pressure of 90/60 mmHg, and a respiratory rate of 20/min. The primary doctor treated him with a 0.9%NaCl total of one liter, ceftriaxone 2 g, and clindamycin 600 mg intravenous infusion. He also exhibited atrial fibrillation and was treated with amiodarone, 150 mg intravenously. The doctor was diagnosed with necrotizing fasciitis on his bilateral hands and referred him to Phramongkutklao Hospital for proper treatment. At the emergency room, his physical examination was remarkable with a drowsy man with a temperature of 37.9°C, a pulse rate of 130/min, and blood pressure of 102/62 mmHg. His conjunctivae were pale without icteric sclerae. The abdominal examination was normal. The examination of the upper limbs showed erythematous swelling and marked tenderness on the bilateral hands and lower arms, ecchymosis on the bilateral thumbs, and bullae developed on the left thumb after that (Figure 1). He had fully bilateral radial and ulnar arterial pulses. Other physical examinations were unremarkable. Laboratory studies demonstrated hemoglobin of 12 g/dL, a white blood cell count of 3,000/μL, with 79.9% neutrophils, 13.8% lymphocytes, and platelets of 105,000/μL. His liver function test showed total bilirubin 1.30 mg/dL, direct bilirubin 0.1 mg/dL, total protein 7.3 g/dL, albumin 4.4 g/dL, aspartate transaminase (AST) 39 U/L, alkaline transaminase (ALT) 41 U/L, and alkaline phosphatase (ALP) 52 U/L. Urgent bilateral hand radiography revealed no evidence of subcutaneous emphysema. The orthopedic surgeon was consulted and an emergency fasciotomy on bilateral hands was immediately performed. Tissue for gram stain showed gram-negative bacilli (Figure 2). After the operation, the hemodynamics stabilized. The patient was admitted for continuous intravenous antibiotics and switched to ciprofloxacin 400 mg intravenous every 12 hours and doxycycline 100 mg orally twice a day after breakfast and dinner for 14 days. Debridement was performed twice after admission on the fifth and eighth day of admission and then he was discharged from the hospital after the wound improved.

## Discussion

*Vibrio vulnificus* is a halophilic gram-negative bacilli found in coastal waters. Ang Sila Coast, Chon Buri Province, Thailand indicated *V. vulnificus* population density was 22% of oyster samples during the rainy season but not found during the summer months [9]. Infection by *V. vulnificus* occurs through ingestion of contaminated food and bacterial translocation in the gastrointestinal mucosa. The clinical presentations include abdominal pain, nausea or vomiting, myalgia, and fever. Bullous skin lesions on the extremities are present, as well as disseminated infection [10].

Necrotizing fasciitis is an aggressive subcutaneous infection along the superficial fascia, which comprises all the tissues between the skin and underlying muscles. The usual organisms are *Streptococcus pyogenes*, *Staphylococcus aureus*, *V. vulnificus*, *Aeromonas hydrophila*, and anaerobic streptococci [11].

The diagnosis of necrotizing fasciitis may not occur on the first presentation. The patient may present cellulitis. Computed tomography (CT) or magnetic resonance imaging (MRI) showed nonspecific changes in edema along the fascial plane and accumulation of fluid in the affected tissues [11].

*Vibrio vulnificus* necrotizing fasciitis, after being exposed to seafood, especially oysters, has shown an increasing prevalence and has been observed among patients with cirrhosis, iron overload states, chronic renal insufficiency, malignancy, HIV status, and immunosuppressive medications [1,3,4,6,9-13]. *V. vulnificus* or *V. parahaemolyticus* was found in 100% of oysters in the Gulf of Mexico [3], with an increased concentration in seasonally warm waters [14], while *V. vulnificus* was found in 10% of crabs. Over 95% of seafood-related deaths have been reported in the US [15].

Due to the severity of *V. vulnificus* necrotizing fasciitis, early diagnosis and treatment are necessary because of high mortality when treatment is delayed [6]. Surgical intervention is the primary treatment in cases of necrotizing fasciitis when the clinical signs are suspected, followed by appropriate intravenous antibiotics, especially combined intravenous doxycycline plus ceftriaxone or cefotaxime [11] or third-generation cephalosporins plus fluoroquinolones [16,17].

This case exhibited some classic appearances associated with *V. vulnificus*, i.e., soft tissue infection after being pinched by mud crabs. Although he did not present any related risk factors for *V. vulnificus* infection, maybe his case needed further investigation. In a related study, a patient receiving an operation within 12 hours after the onset of symptoms exhibited a higher survival rate than those with a delayed operation [18]. Combining intravenous antibiotics with third-generation cephalosporins and fluoroquinolones was preferred in this condition [16,17,19].

## Conclusions

*Vibrio vulnificus* necrotizing fasciitis constitutes an emergency condition. Patients with underlying liver disease developed septicemia and soft tissue infections after ingesting or being exposed to raw seafood. Greater awareness of *V. vulnificus* infection should be made and proper management for surgical and medical treatment should be performed. Combining intravenous antibiotics with third-generation cephalosporins and fluoroquinolones was preferred.
